# Successful management of delayed post-PCNL renal pseudoaneurysm using a percutaneous endoscopic transurethral resection system: A case report

**DOI:** 10.1016/j.eucr.2026.103528

**Published:** 2026-07-01

**Authors:** Mehdi Sotoudeh, Seyedhossein Rabani, Behzad Narouie, Ali Adib

**Affiliations:** a Department of Endourology, Day General Hospital, Tehran, Iran; b Urology and Nephrology Research Center (UNRC), Research Institute for Urology and Nephrology (UNRI), Shahid Beheshti University of Medical Sciences (SBMU), Tehran, Iran; c Department of Urology, Zahedan University of Medical Sciences, Zahedan, Iran; d Urology and Nephrology Research Center (UNRC), Center of Excellence in Urology, Shahid Labbafinajad Hospital, Shahid Beheshti University of Medical Sciences (SBMU), Tehran, Iran; e Urology Department, Shiraz University of Medical Sciences, Shiraz, Iran

## Abstract

Renal artery pseudoaneurysm is a rare but potentially life-threatening complication following percutaneous nephrolithotomy (PCNL). We report a female who developed massive gross hematuria after an uncomplicated right PCNL. Computed tomography angiography revealed a renal artery pseudoaneurysm, and selective angioembolization failed to achieve definitive hemostasis. As a salvage approach, percutaneous endoscopic intervention was performed. Under direct intrarenal visualization, the actively bleeding arterial branch was identified and successfully coagulated, achieving complete hemostasis. The postoperative course was uneventful with no recurrence of hematuria. This case demonstrates that percutaneous endoscopic coagulation can be an effective alternative for refractory post-PCNL renal pseudoaneurysms.

## Highlights

• **Clinical Challenge:** Describes a rare, life-threatening case of delayed renal artery pseudoaneurysm presenting as massive gross hematuria 40 days after PCNL.
• **Failed Gold Standard:** Reports a scenario where standard selective arterial angioembolization proved ineffective at achieving definitive hemostasis.
• **Innovative Salvage Technique:** Demonstrates the successful use of a standard monopolar transurethral resection (TUR) system for direct intrarenal coagulation of the bleeding vessel.
• **Precision & Preservation:** Emphasizes that this endoscopic approach allows for precise, direct visualization and targeted vascular sealing, effectively preserving renal parenchyma and function.
• **Clinical Significance:** Proposes this technique as a viable, effective alternative for managing complex post-PCNL vascular complications when conventional endovascular methods fail, preventing the need for more invasive procedures or nephrectomy.

## 1 Introduction

Percutaneous nephrolithotomy (PCNL) is the standard of care for the management of large and complex renal calculi and is associated with high stone-free rates.^1^^,^^2^ Despite its overall safety profile, PCNL carries a risk of hemorrhagic complications, which remain among the most serious adverse events. While most bleeding occurs intraoperatively or in the early postoperative period, delayed hemorrhage is uncommon and is typically attributable to renal vascular injuries, including arteriovenous fistulas and renal artery pseudoaneurysms.

Renal artery pseudoaneurysm, though rare, represents a critical and potentially life-threatening complication of percutaneous nephrolithotomy if not promptly and effectively managed.^3^ This condition typically arises from arterial wall injury during the procedure, leading to a pulsatile hematoma contained by perirenal soft tissue rather than a true vessel wall.^4^ The clinical presentation often involves delayed gross hematuria, flank pain, and, in severe cases, hypovolemic shock, necessitating urgent diagnostic imaging such as CT angiography for confirmation.^4^^,^^5^ Management strategies for these pseudoaneurysms commonly involve percutaneous endovascular techniques, including super selective embolization, to occlude the affected vessel and prevent further hemorrhage.^6^

Herein, we describe a patient who developed pseudoaneurysm following PCNL, which was successfully managed with percutaneous endoscopic treatment.

## 2 Case presentation

A 42-year-old female presented to the emergency department with massive gross hematuria approximately 40 days following an uncomplicated right percutaneous nephrolithotomy procedure. Upon admission, the patient was hemodynamically unstable, exhibiting signs of significant blood loss. Laboratory findings revealed a substantial drop in hemoglobin levels, necessitating the transfusion of 11 packed red blood cell units in the whole course of admission.

**Fig. 1:**
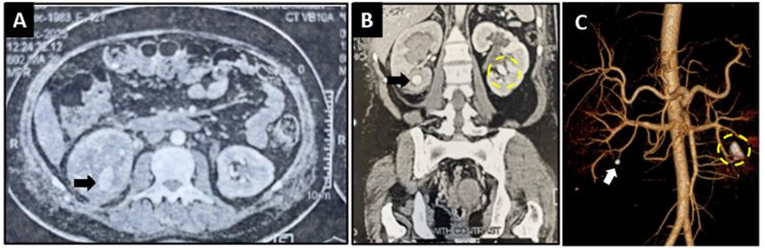
(A) Axial post-contrast abdominal CT scan demonstrates right side renal artery pseudoaneurysm (black arrows). (B) Coronal post-contrast image also shows the pseudoaneurysm (black arrows) and a non-obstructive stone is noted at lower pole of left kidney (dotted yellow circle). (C) In 3D reconstructed image, angio-embolization was done and embolization coil is shown (white arrow) as well as the mentioned left renal stone (dotted yellow circle).

After initial stabilization, computed tomography angiography was performed to identify the source of bleeding, revealing a renal artery pseudoaneurysm (Fig. 1). Given the severity of the hemorrhage and hemodynamic instability, arterial angioembolization was attempted. However, this intervention proved unsuccessful in achieving definitive hemostasis. Due to the persistent, massive gross hematuria and the failure of angioembolization, a decision was made for immediate surgical intervention.

## 3 Surgical procedure

**Fig. 2:**
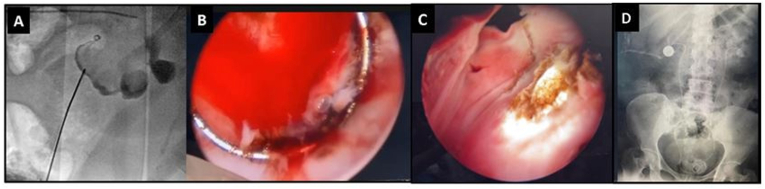
(A)Access to the lower pole of the right kidney to localize the bleeding source. (B) Hemostasis under continuous direct endoscopic visualization using a standard monopolar transurethral resection system. (C) Nephroscopic view of the aneurysmal cavity. (D) Balloon nephrostomy inflated to 3mL with water and contrast material.

The patient was positioned prone under general anesthesia. The collecting system was opacified through retrograde contrast injection under fluoroscopy to identify the calyx containing pseudoaneurysm. Percutaneous antegrade access to the target calyx was then obtained under fluoroscopic guidance. Preoperative imaging was correlated with intraoperative fluoroscopic findings to identify the target calyx and to selecting the most appropriate access tract accordingly. Upon endoscopic exploration, a renal artery pseudoaneurysm was identified. After identification of the pseudoaneurysm and evacuation of intrarenal clots, the bleeding source was clearly visualized as an actively bleeding arterial branch within the pseudo aneurysmal cavity. Under continuous direct endoscopic visualization, a standard monopolar transurethral resection system, routinely used for transurethral resection of bladder tumors (TURB), was introduced into the renal collecting system (Fig. 2).

Hemostasis was achieved using a TURB loop electrode, applying a technique analogous to conventional bladder tumor resection, with appropriate adaptation to the intrarenal anatomy. Controlled and stepwise monopolar coagulation was performed directly on the bleeding arterial branch, starting from the base of the vessel to ensure complete vascular sealing, followed by coagulation of the pseudoaneurysm wall to prevent recurrent hemorrhage. The procedure was conducted carefully to maintain optimal endoscopic visibility and avoid excessive thermal injury to the surrounding renal parenchyma.

The coagulations were performed using a Karl Storz monopolar TURB resectoscope system (Karl Storz SE & Co. KG, Tuttlingen, Germany). Upon completion, the treated area was thoroughly inspected, confirming complete hemostasis with no residual active bleeding or vascular oozing.

Antegrade DJ stent 4.8 F was inserted. Following successful hemostasis, a 16F Foley catheter was used as a temporary nephrostomy and tamponade device (Fig. 2). The catheter was introduced through the established percutaneous nephrostomy tract into the target calyx within the collecting system. A small amount of contrast was injected under fluoroscopic guidance to confirm accurate intrarenal positioning of the catheter tip and balloon. Subsequently, the balloon was inflated with 3 mL of water and positioned to achieve local tamponade adjacent to the lesion, while ensuring adequate drainage.

The patient's postoperative course was uneventful, and the balloon nephrostomy tube was removed two days after the procedure. The patient was subsequently discharged with stable vital signs and no further episodes of hematuria. DJ stent was removed under local anesthesia two weeks later.

## 4 Discussion

The described case illustrates the critical importance of effective strategies for managing delayed post-PCNL hemorrhage, particularly when initial endovascular interventions prove insufficient.^7^ Renal artery pseudoaneurysm, a rare but potentially life-threatening vascular complication, occurs in approximately 0.6%–1% of PCNL cases.^1^^,^^8^ This injury originates from damage to the arterial wall during the procedure, leading to a contained hematoma that eventually communicates with the arterial lumen, often presenting as delayed gross hematuria.^4^^,^^5^

The patient presented in this report developed massive gross hematuria approximately 40 days post-PCNL. This significantly delayed presentation is noteworthy, as delayed bleeding typically manifests within the first three weeks following surgery, underscoring the unpredictable nature of such vascular complications.^7^ Accurate diagnosis, primarily through computed tomography angiography, is crucial for optimal management, as it enables localization of the pseudoaneurysm and informs targeted therapeutic interventions.^5^^,^^9^

The primary treatment for post-PCNL renal artery pseudoaneurysm is selective trans-arterial embolization, widely considered the gold standard due to its high efficacy, kidney-sparing potential, and minimal invasiveness, with success rates of 85%–100%.^5^^,^^8^^,^^10^^,^^11^ Despite its overall success, angioembolization can occasionally fail to achieve definitive hemostasis, as observed in this case. Factors contributing to embolization failure can include technical difficulties, the complex anatomy of the renal vasculature, the size or location of the pseudoaneurysm, or even patient-specific contraindications like contrast hypersensitivity.^7^^,^^12^ The failure of initial angioembolization necessitates consideration of alternative, often more invasive, salvage strategies to prevent further blood loss, which could otherwise lead to significant morbidity or even nephron loss and, in severe cases, nephrectomy.^7^

This case uniquely demonstrates the successful application of percutaneous endoscopic treatment utilizing a standard monopolar transurethral resection system in a scenario where angioembolization proved unsuccessful. This approach allowed for direct intrarenal visualization of the actively bleeding arterial branch within the pseudoaneurysmal cavity. The meticulous monopolar coagulation of the bleeding vessel and the pseudoaneurysm wall, adapted from conventional bladder tumor resection techniques, provided precise hemostasis under direct endoscopic control. This direct, targeted intervention provides advantages over blind or less precise methods by reducing recurrent bleeding risk.

One potential concern associated with the use of monopolar electrocautery in the treatment of renal pseudoaneurysms is the risk of irrigant absorption through the injured vascular bed, which may theoretically result in dilutional hyponatremia. In the present case, no clinical evidence of significant fluid absorption was observed. The procedure was completed within approximately 45 minutes using an estimated 8 L of irrigation fluid, which may have limited the risk of substantial intravascular absorption. Nevertheless, awareness of this potential complication and appropriate perioperative monitoring should be considered when applying this technique, particularly during prolonged procedures or in cases involving extensive vascular exposure.

While other salvage options for failed angioembolization have been described, including percutaneous re-surgical removal of the pseudoaneurysm using a nephro-grasper^8^ or nephroscopy with tamponade,^7^ the described endoscopic coagulation technique provides a solution, preserving renal parenchyma and function. The positive outcome, characterized by complete hemostasis and an uneventful post-operative course, highlights this method as a viable and effective alternative for managing complex post-PCNL pseudoaneurysms. This case offers insights into managing complex vascular complications after PCNL, highlighting the need for strategies and advanced endourological skills when standard methods fail.

## 5 Conclusion

This case report highlights an innovative endoscopic approach for managing complex renal pseudoaneurysms that are refractory to conventional angioembolization.

## CRediT authorship contribution statement

**Mehdi Sotoudeh:** Conceptualization, Data curation, Writing – original draft, Writing – review & editing. **Seyedhossein Rabani:** Conceptualization, Data curation, Writing – review & editing. **Behzad Narouie:** Conceptualization, Writing – original draft. **Ali Adib:** Conceptualization, Data curation, Validation, Writing – original draft.

## References

[1] Anand A., Manhas A. (2020 Jul 18). Post PCNL renal pseudoaneurysm and AV fistula: single institution analysis. Int Arch Urol Complic.

[2] Poudyal S. (2022 Jan 1). Current insights on haemorrhagic complications in percutaneous nephrolithotomy. Asian J Urol.

[3] Mavuduru R.M., Mohd Ziauddin S.A., Bora G.S., Gorsi U. (2024 Apr 19). Renal artery pseudoaneurysm following robot assisted nephron sparing surgery: two case reports. J Med Case Rep.

[4] Deng X.X., Zhang W., Fu D., Fu B. (2022 May 12). Renal pseudoaneurysms after flexible ureteroscopy and holmium laser lithotripsy: a case report. Front Surg.

[5] Tadayon N., Refaei M., Zarrintan S., Shahsavari S., Najari D., Sheikhzadeh M. (2024 Mar 13). Post-percutaneous nephrolithotomy pseudo aneurysm formation treated by coil embolization; A study of seven cases. J Cardiovasc Thorac Res.

[6] Gooran S., Javid A.M., Pourmand G. (2018 Feb 1). Delayed hemorrhage in kidney transplantation: a life-threatening condition. Int J Organ Transplant Med.

[7] Nouralizadeh A., Aslani A., Ghanaat I., Bonakdar Hashemi M. (2020 Sep 1). Percutaneous endoscopic treatment of complicated delayed bleeding postpercutaneous nephrolithotomy: a novel suggestion. J Endourol Case Rep.

[8] Nouralizadeh A., Rostaminejad N., Radpour N., Momeni H., Narouie B., Dadpour M. (2023 Sep 1). Percutaneous re-surgical approach for delayed bleeding caused by pseudoaneurysm following percutaneous nephrolithotomy. Urol Case Rep.

[9] Lasmanovich R., Mahmud H., Khaitovich B. (2025 Mar 18). Early angiography improves postoperative clinical outcomes compared to delayed angiography among patients with vascular pathologies following partial nephrectomy. World J Urol.

[10] Pakdel A., Asgari F., Bahri R.A., Aghamir S.M. (2024 Mar 12). Segmental artery angioembolization as an efficient treatment modality for delayed hematuria with normal angiography: two case reports. J Med Case Rep.

[11] Ngo T.C., Lee J.J., Gonzalgo M.L. (2010 Nov). Renal pseudoaneurysm: an overview. Nat Rev Urol.

[12] Mao Q., Wang C., Chen G., Tan F., Shen B. (2019 Nov). Failure of initial superselective renal arterial embolization in the treatment of renal hemorrhage after percutaneous nephrolithotomy: a respective analysis of risk factors. Exp Ther Med.

